# Case Report: A very rare case of a pleural effusion revealing multiple myeloma

**DOI:** 10.12688/f1000research.133007.1

**Published:** 2023-05-09

**Authors:** Selsabil Daboussi, Asma Saidane, Samira Mhamdi, Marwa Kacem, Samia Essbaa, Chiraz Aichaouia, Hela Ghedira, Faten Gargouri, Issam Msakni, Zied Moatemri

**Affiliations:** 1 Department of Pneumology, Military Hospital, Tunis, 1008, Tunisia; 2 Faculty of Medicine, University of Tunis El Manar, Tunis, 1007, Tunisia; 3 Department of Hematology, Military Hospital, Tunis, 1008, Tunisia; 4 Pathology Departmeny, Military Hospital, Tunis, 1008, Tunisia

**Keywords:** Myeloma, pleura, malignant, thoracoscopy

## Abstract

Multiple myeloma is a common malignant bone-based disease. Pleural effusions reported in these patients remain rare. It is commonly due to congestive heart disease, pulmonary embolism, nephrotic syndrome or a second neoplasia. The true myelomatous pleural effusion resulting from a direct tumoral invasion of the pleural are extremely rare. We report here the case of a massive pleural effusion revealing multiple myeloma in a 71-year-old patient. The chest ultrasound showed a massive pleural effusion in the left side with a multinodular thickening of the pleura. The medical thoracoscopy showed a grape-cluster appearance. The diagnosis was made by pleural guided biopsy revealing abnormal plasma cells with an intense positive CD 138 (plasma cell marker) and MUM1 (multiple myeloma oncogene1) staining with a light kappa chain in the protein electrophoresis associated with a myeloma. Unfortunately, our patient died one month after the initial diagnosis. We present also a review of the recent literature in order to highlight the clinical presentations of the myelomatous pleural effusion, the diagnostic tools, the therapeutic strategies as well as the outcomes.

## Introduction

Multiple myeloma is a common malignant disease due to a proliferation of an abnormal clone of plasma cells associated with a monoclonal protein or a light chain in the serum or in the urine. ^1^ However, the myelomatous pleural invasion is very rare. We report a case of a massive left-sided myelomatous pleural effusion revealing the disease. The diagnosis was made by the identification of abnormal cells with an intense positive staining to CD138 (plasma cell marker) and a kappa light chain in the protein electrophoresis. We also present a review of the current literature in order to highlight the clinical presentation, the diagnostic tools, the therapeutic approaches and the outcomes.

## Case report

A 71-year-old woman was admitted in our Department of Pneumology in November 2022 for acute respiratory failure. She complained of dyspnoea occurring at the slightest effort (III NYHA) associated with an important deterioration of her general status (weight loss, asthenia, anorexia). She has previously been treated for cardiac disease (Plavix 75 mg per day, Aspegic 100 mg/day, Statinor 80 mg per day, Sotalol 160 mg per day), atrial fibrillation (Cordarone 200 mg per day (5 days per week) with a curative dose anticoagulant treatment (Rivaroxaban 20 mg/day)) and for psychiatric depressive disorders (with treatment interruption). She is a house-wife and is Caucasian. With regards her habits, she does not smoke and does not drink alcohol. There was no significant past family medical history nor environmental exposure, especially to asbestos.

Physical examination found a deteriorated general status (performance status = 3). She was afebrile. Her pulse rate was 78 ppm. Her blood pressure was 130/70 mmHg. Her respiratory rate was 24 cpm. She did not display any sign of respiratory distress. The breath sounds were abolished in the left side. The oxygen desaturation was (89%) on room air. The electrocardiogram was normal.

Lab tests showed hypochromic microcytic anaemia (Hb level = 9.9 g/dl) and hypercalcemia (calcium level = 2.96 mmol/l).

**Figure 1.**
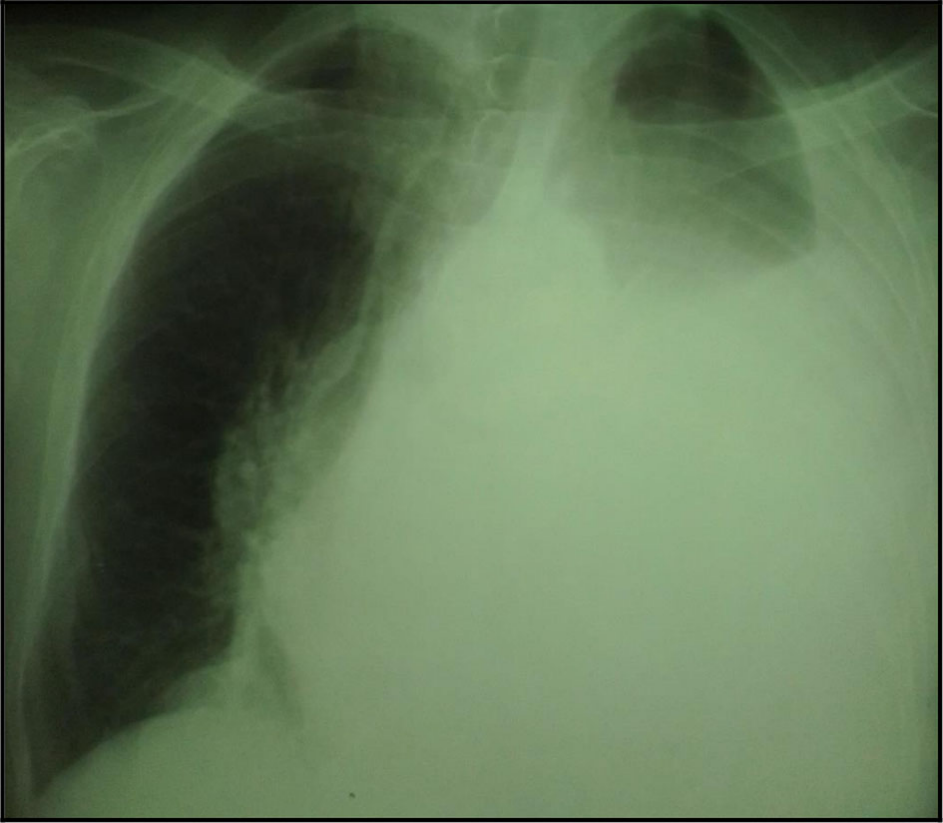
Chest X-ray showing a pleural opacity in the left chest repressing the trachea and the mediastinum.

**Figure 2.**
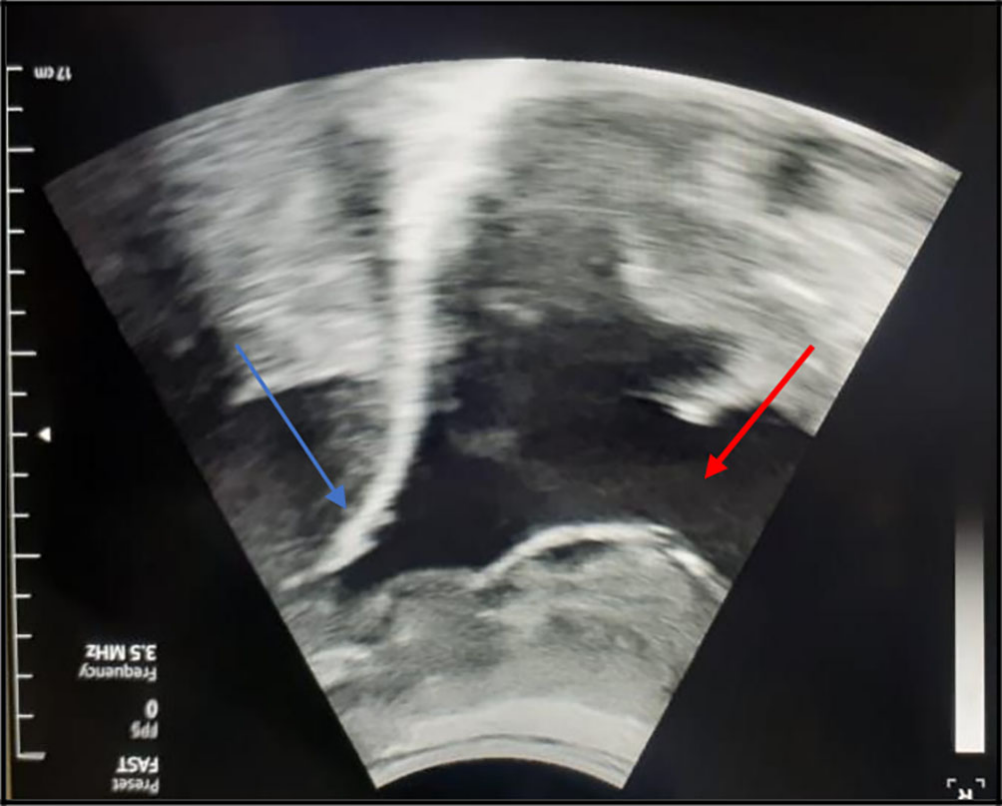
The chest ultrasound showed a massive, anechoic, free pleural effusion (red arrows) in the left side associated with a multinodular pleura (blue arrows).

**Figure 3.**
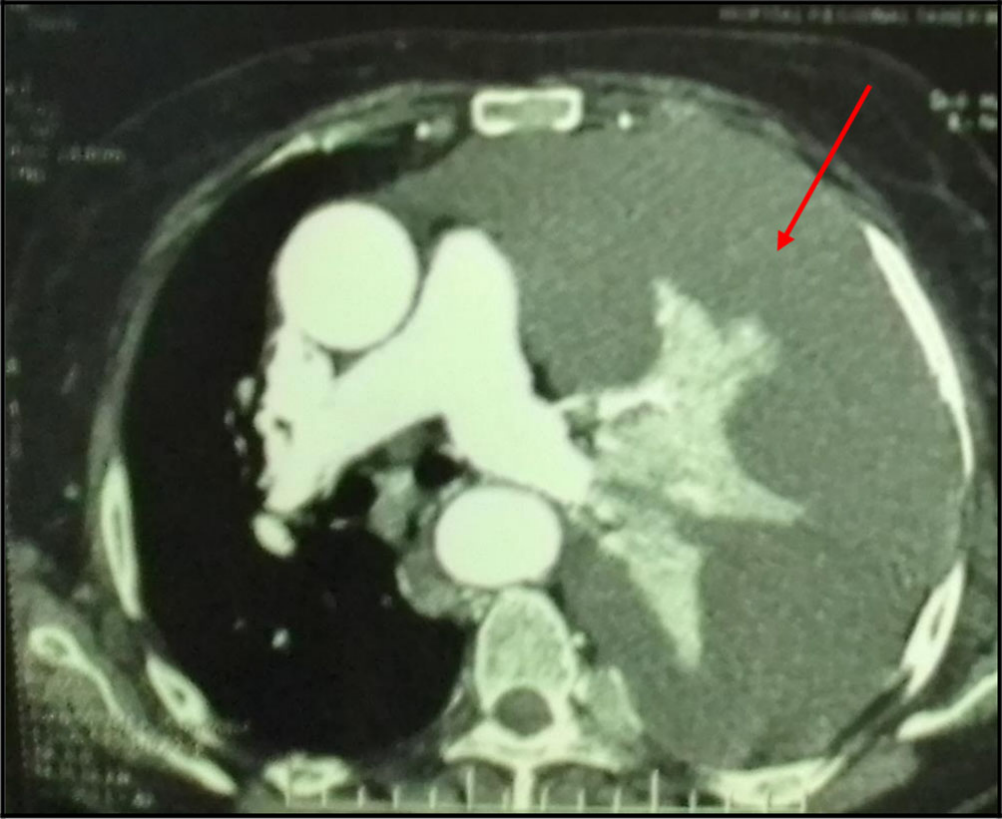
A mediastinal chest CT section revealing a massive pleural effusion in the left side (red arrows).

The chest X-ray showed left pleural opacity with signs of compression ( Figure 1). So, an exploratory and evacuating ultrasound-guided pleural puncture was immediately performed. The thoracic ultrasound showed a massive anechoic, free left pleural effusion associated with pleural nodules ( Figure 2). Analysis of the pleural fluid showed a serohaematic exudative fluid with a predominantly lymphocyte formula (80%). A Gram stain fast bacilli (AFB) stain and bacterial and tuberculosis cultures for were all negative. Therefore, a malignant origin was suspected. The chest CT-scan revealed a left-sided malignant pleural effusion associated with mediastinal adenopathy, extended secondary bone lesions and subcutaneous lesions ( Figure 3). Additionally, the echocardiography was normal.

**Figure 4.**
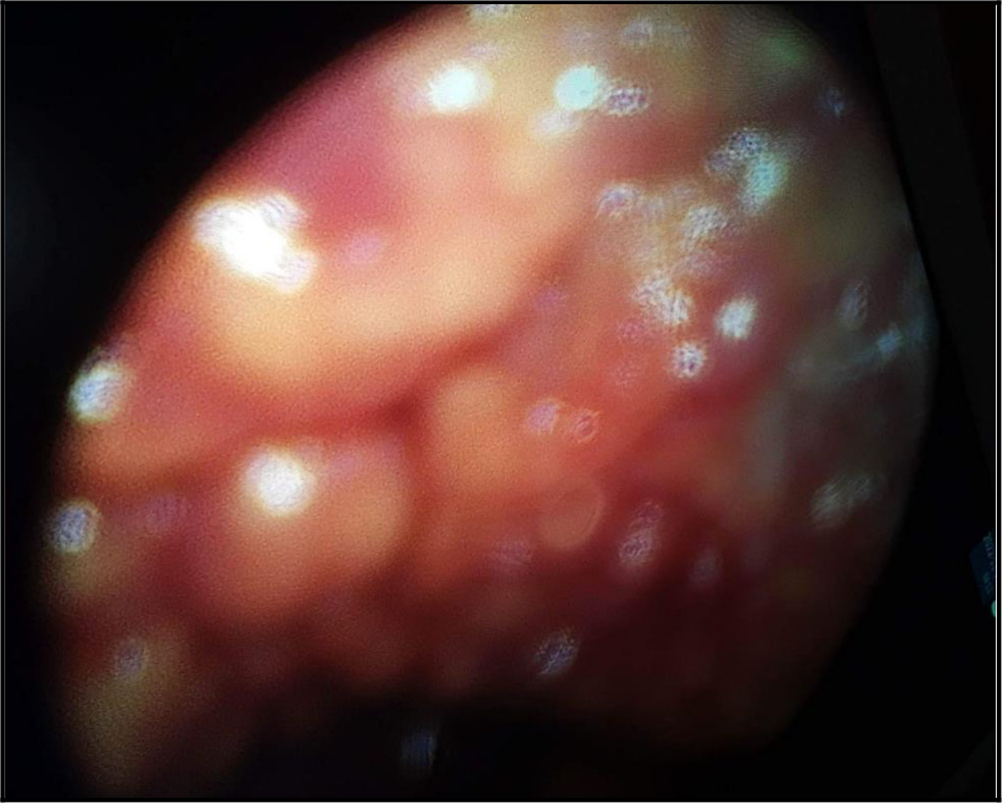
The ‘grape-cluster’ like aspect of the pleura as seen in medical thoracoscopy performed in our Department of Pneumology.

A medical thoracoscopy was performed 9 days after her admission in our department. It showed a multinodular pleura with a “bunch of grapes” sign ( Figure 4). It allowed guided pleural biopsies as well as chemical pleurodesis in order to prevent the pleural effusion recurrence.

Furthermore, the patient reported an acute neck pain associated with tetraparesis 2 weeks later, during her hospital stay. A spinal MRI showed a tumoral process infiltrating the axis resulting in a spinal cord compression. There was not any sign of spinal suffering. The MRI also revealed diffuse bone involvement with some nodular lesions. So, emergency decompressive radiotherapy was performed as a salvage therapy with immobilization of the cervical spine.

**Figure 5.**
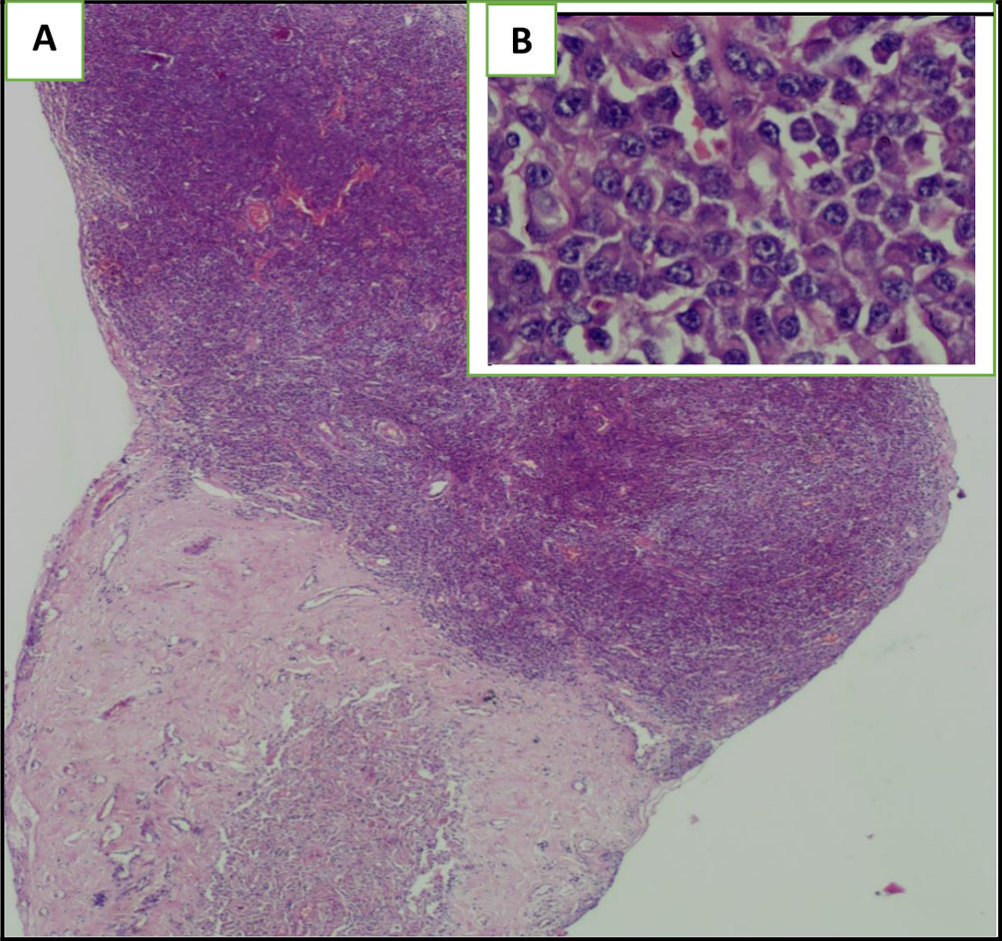
The haematoxylin eosin (HE) staining illustrates the presence of an undifferentiated abnormal rounded tumoral cells: (A): Envision (HE)*2, (B): Envision (HE)*40.

**Figure 6.**
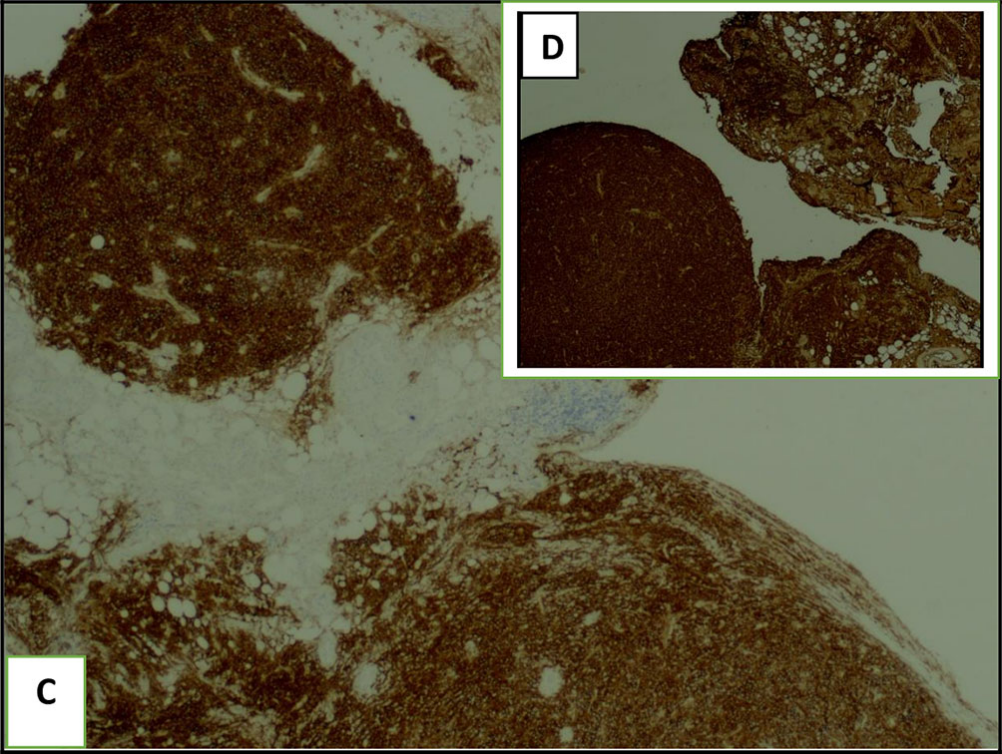
The pleural guided biopsy block with immunochemistry shows abnormal plasma cells with an intense positive CD138 staining (C) and a light kappa chain (D).

The histological examination of the pleural biopsy showed abnormal round tumoral cells ( Figure 5). Immunochemistry revealed an intense and diffuse positive staining for CD138 (plasma cell marker) and MUM1 (multiple myeloma oncogene 1) with a kappa light chain ( Figure 6) suggesting a myelomatous pleural effusion (MPE). The serum protein electrophoresis showed a monoclonal peak at the kappa chain gammaglobulin region. A 24-hour proteinuria was negative. Additionally, the electrophoresis and immunofixation of urinary proteins was normal.

The diagnosis of myelomatous pleural effusion was made given: the presence of abnormal plasma cells with a positive staining CD 138 and MUM1 in the pleural biopsy, the presence of a kappa-light chain in the protein electrophoresis and the presence of a multiple myeloma confirmed histologically with the presence of the CRAB criteria (hypercalcemia, anaemia, presence of several osteolytic lesions on imaging). Unfortunately, our patient died one month after the initial diagnosis because of a fatal progression of her disease.

## Discussion

Multiple myeloma is a bone-based disease, first described in Egyptian mummies. The median age of occurrence is 69 years and it occurs mainly in men. ^2^ It can invade many organs such as the chest, the bones, and the skin. However, the pleural effusions reported in these patients remain rare (6%). ^3^ Moreover, the true myelomatous pleural effusion (MPE) is extremely rare. It occurs in less than (1%) of cases. It is more common in IgG Myeloma types (40%). ^4^

The diagnosis may be challenging because the symptoms are not specific. In fact, the patients can present with a wide spectrum of clinical features. They report typically hematologic symptoms due to the bone marrow invasion (anaemia, bleeding, infections) and bone lesions (hypercalcemia). They may also report symptoms due to the infiltration of the surrounding organs. ^5^^,^^6^ Our patient complained of dyspnoea on exertion associated with an important deterioration of the general status.

Pleural effusion associated with multiple myeloma may be due to a congestive heart failure. It is often seen in secondary amyloidosis. It may result either from hyperviscosity or myocarditis, commonly seen in these patients especially after the treatment onset. ^1^^,^^7^ Pulmonary embolism should be also considered because of the advanced age of the patients, the comorbidities and the current neoplasia. ^8^ Third, the nephrotic syndrome and the chronic renal failure may be responsible of a concomitant pleural effusion. ^9^ Pulmonary embolism was ruled out by the chest CT scan in our case. The kidney function and 24-hour proteinuria were normal. The pleural effusion may result from a second concomitant neoplasia especially a carcinoma (breast cancer in women, lung cancer in men) or a mesothelioma. ^1^^,^^10^ This should be considered in the differential diagnosis of a malignant pleural effusion. Our patient was not a smoker and the breast exam was normal.

The diagnosis was very challenging. In fact, we initially suspected an advanced stage lung carcinoma giving the chest CT scan’s findings, or a mesothelioma due to the multinodular aspect of the pleura in the chest ultrasound and “the grape-cluster” appearance as seen in thoracoscopy. Histology helped us to assess the right diagnosis.

It is worth mentioning that true myelomatous pleural effusion is extremely rare. It may result either from: a direct extension from an adjacent skeletal or lung lesions; a direct tumoral invasion of the pleura; a lymphatic obstruction by mediastinal adenopathy; or it may be due to the presence of a multiple myeloma in the chest wall, as seen in our patient. ^11^^,^^12^

The diagnosis requires the presence of a monoclonal protein or a light chain in the pleural fluid, abnormal plasma cells with an intense staining CD138. ^13^^–^^16^ Medical thoracoscopy is actually recommended in the case of such malignant pleural effusion. In fact, it allows pleural guided-biopsy with a good-quality sampling, pleural fluid drainage as well as pleurodesis in order to prevent the recurrences during the follow-up. ^8^^,^^17^^,^^18^ In our case, medical thoracoscopy showed a multinodular pleura with “a buck of grapes” like aspect. We decided to perform chemical pleurodesis given that most of the reported cases had a high rate of recurrence of the pleural effusion. Flow cytometry may be a useful tool to assess the diagnosis. ^19^

Another interesting aspect of this case is that the pleural effusion revealed the disease. It is well known that the pleural effusion is a late manifestation during the natural history of the multiple myeloma associated with a poor prognosis. ^20^ The median survival does not exceed four months according to literature, despite an aggressive therapeutic approach. ^21^

Treatment is based on a combination of chemotherapy (Adriamycin, vincristine, doxorubicin …) or palliative radiotherapy as a salvage therapy. A new drug has been used (Bortezomib) which is a proteasome inhibitor with good results. ^22^ Stem cell transplantation can be also considered. ^23^ Unfortunately, the patient’s response is often transient with a high rate of tumoral recurrence. ^11^^,^^24^

## Conclusion

To conclude, we report a very rare case of a massive pleural effusion revealing multiple myeloma. This should be considered in the differential diagnosis of a malignant pleurisy. Medical thoracoscopy is a mainstream exam in order to assess the diagnosis and to prevent the recurrence. Further studies using flow cytometry and cytogenetic analysis are required. The new therapeutic approaches using target therapy drugs seem to be very interesting pathways.

## Consent

Written informed consent for publication of their clinical details and images was obtained from the family of the patient.

## Data Availability

All data underlying the results are available as part of the article and no additional source data are required.
