# Case Report: A very rare case of a Pleural Effusion revealing Multiple Myeloma

**DOI:** 10.12688/f1000research.133007.3

**Published:** 2023-11-03

**Authors:** Selsabil Daboussi, Asma Saidane, Samira Mhamdi, Marwa Kacem, Samia Essbaa, Chiraz Aichaouia, Hela Ghedira, Faten Gargouri, Issam Msakni, Zied Moatemri

**Affiliations:** 1 Faculty of Medicine, University of Tunis El Manar, Tunis, 1007, Tunisia; 2 Department of Pneumology, Military Hospital, Tunis, 1008, Tunisia; 3 Department of Hematology, Military Hospital, Tunis, 1008, Tunisia; 4 Pathology Departmeny, Military Hospital, Tunis, 1008, Tunisia

**Keywords:** Myeloma, pleura, malignant, thoracoscopy

## Abstract

Multiple myeloma is a common malignant bone-based disease. Pleural effusions reported in these patients remain rare. It is commonly due to congestive heart disease, pulmonary embolism, nephrotic syndrome or a second neoplasia. The true myelomatous pleural effusion resulting from a direct tumoral invasion of the pleural are extremely rare. We report here the case of a massive pleural effusion revealing multiple myeloma in a 71-year-old patient. The chest ultrasound showed a massive pleural effusion in the left side with a multinodular thickening of the pleura. The medical thoracoscopy showed a grape-cluster appearance. The diagnosis was made by pleural guided biopsy revealing abnormal plasma cells with an intense positive CD 138 (plasma cell marker) and MUM1 (multiple myeloma oncogene1) staining with a light kappa chain in the protein electrophoresis associated with a myeloma. Unfortunately, our patient died one month after the initial diagnosis. We present also a review of the recent literature in order to highlight the clinical presentations of the myelomatous pleural effusion, the diagnostic tools, the therapeutic strategies as well as the outcomes.

## Introduction

Multiple myeloma is a common malignant disease due to a proliferation of an abnormal clone of plasma cells associated with a monoclonal protein or a light chain in the serum or in the urine. ^1^ However, the myelomatous pleural invasion is very rare. We reported a case of a massive left-sided myelomatous pleural effusion revealing the disease. The diagnosis was made by the identification of abnormal cells with an intense positive staining to CD138 (plasma cell marker) and a kappa light chain in the protein electrophoresis. We also presented a review of the current literature in order to highlight the clinical presentation, the diagnostic tools, the therapeutic approaches and the outcomes.

## Case report

A 71-year-old woman was admitted in our department of Pneumology in November 2022 for acute respiratory failure. She complained of dyspnea occurring at the slightest effort (III NYHA) associated with an important deterioration of her general status (weight loss, asthenia, anorexia). She had been previously followed-up for an ischemic cardiac disease (Plavix 75 mg per day, Aspegic 100 mg/day, Statinor 80 mg per day, Sotalol 160 mg per day), an atrial fibrillation (Cordarone 200 mg per day (5 days per week)) under a curative dose anticoagulant treatment (Rivaroxaban 20 mg/day)) and for a psychiatric depressive disorder (with a self-treatment interruption). She was a house-wife. She was a Caucasian. She was not a smoker and did not drink alcohol. There was not any significant past medical family history nor an environmental exposure, especially to asbestos.

The physical examination found a deteriorated general status (performance status = 3). She was afebrile. Her pulse rate was 78 ppm. Her blood pressure was 130/70 mmHg. Her respiratory rate was 24 cpm. She had not any sign of respiratory distress. The breath sounds were abolished in the left side. Her oxygen saturation was (89%) on room air. The electrocardiogram was normal.

Lab tests showed an hypochromic microcytic anemia (Hemoglobin level = 9.9 g/dl) and hypercalcemia (Calcium level = 2.96 mmol/l). The white blood cells (WBC) count was normal (8200/mm ^3^). The lymphocyte count was normal (1400/mm ^3^). The platelet count was high (534000/mm ^3^). The C-reactive protein (CRP) level was high (35 mg/l). The procalcitonin level was normal. The erythrocyte sedimentation rate (ESR) was high (100 mm/hour). The kidney and liver functions were normal.

**Figure 1.**
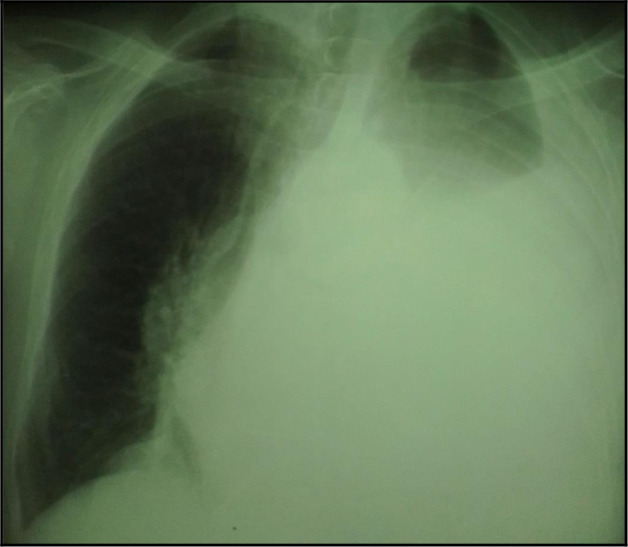
Chest X-ray showing a pleural opacity in the left chest repressing the trachea and the mediastinum.

**Figure 2.**
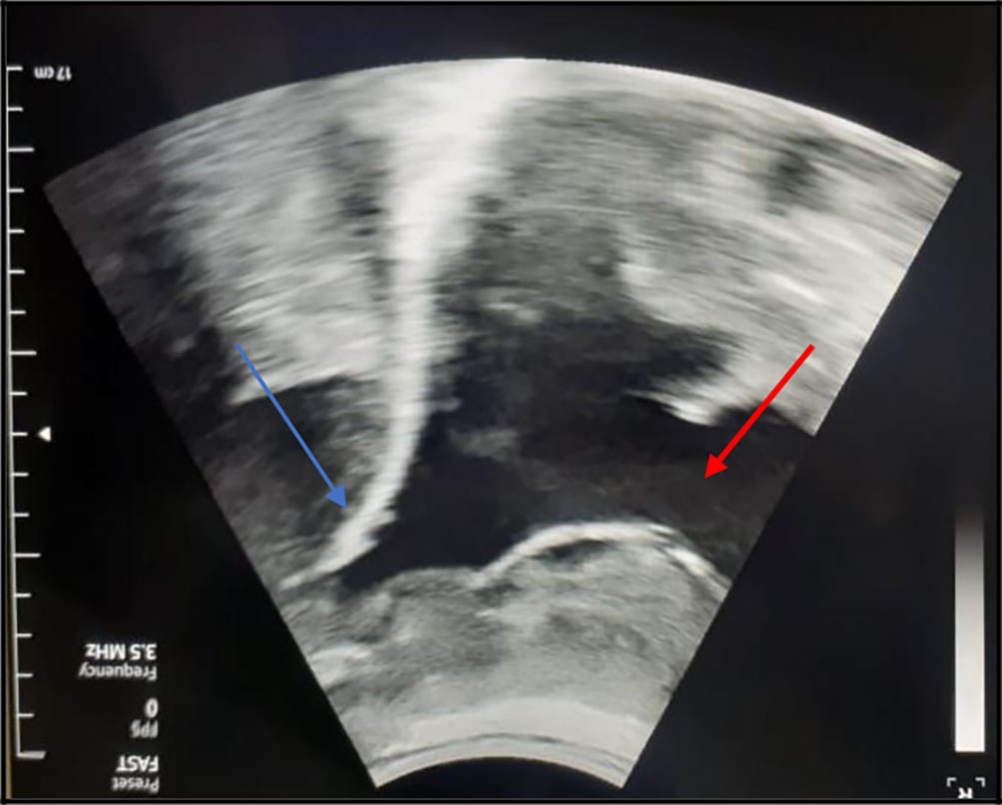
The chest ultrasound showed a massive, anechoic, free pleural effusion (red arrows) in the left side associated with a multinodular pleura (blue arrows).

The chest X-ray showed a left-sided pleural opacity with signs of compression ( Figure 1). So, an exploratory and evacuating ultrasound-guided pleural puncture was immediately performed. The thoracic ultrasound revealed a massive anechoic, free left pleural effusion associated with many pleural nodules ( Figure 2). Therefore, a malignant origin was suspected. The analysis of the pleural fluid showed a serohaematic exudative fluid, with a predominantly lymphocyte formula (80%). A Gram stain fast bacilli (AFB) stain and cultures for (bacterial and tuberculosis) were all negative. We did not find any malignant cell in the pleural puncture cytology.

**Figure 3.**
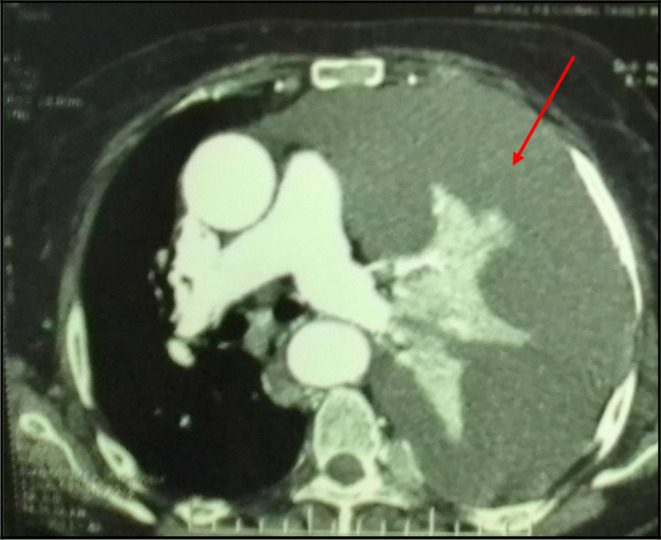
A mediastinal chest CT section revealing a massive pleural effusion in the left side (red arrows).

The Body-scan revealed a left-sided malignant pleural effusion associated with mediastinal lymph nodes. It ruled out a pulmonary embolism. We also noticed extended secondary bone and subcutaneous lesions ( Figure 3). However, it did not show any tumoral involvement of the skull or the pelvis. The echocardiography was normal.

**Figure 4.**
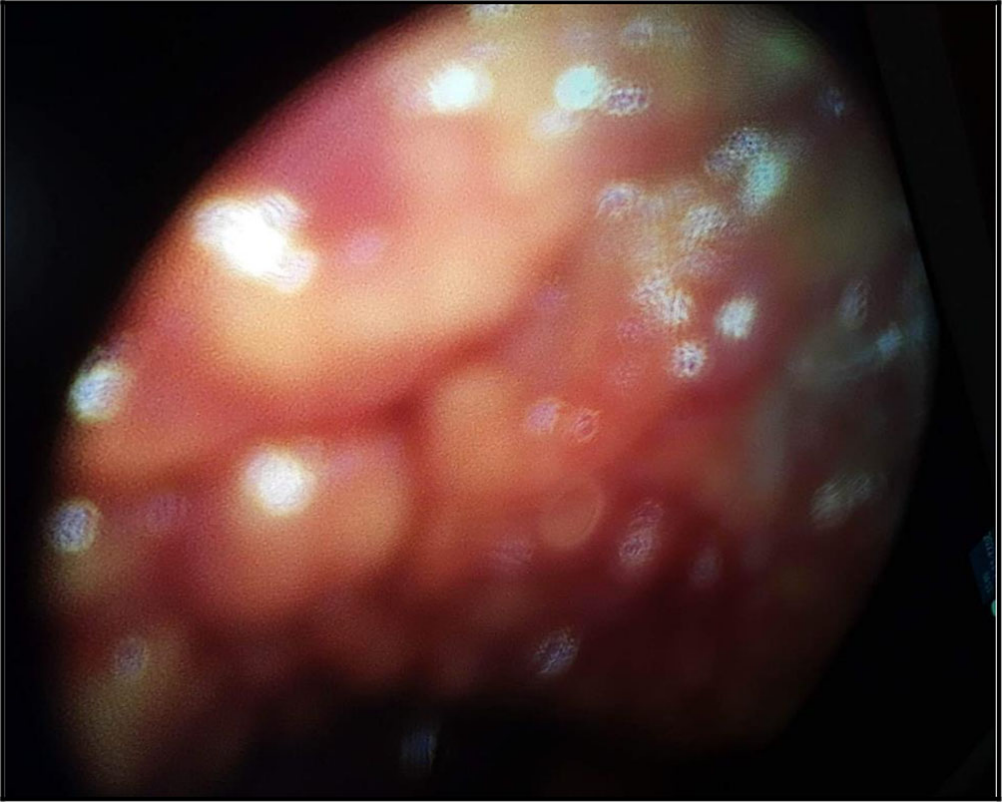
The ‘grape-cluster’ like aspect of the pleura as seen in medical thoracoscopy performed in our department of Pneumology.

Thus, a medical thoracoscopy was performed 9 days after her admission in our department. It showed a multinodular pleura with a “bunch of grapes” aspect ( Figure 4). It allowed guided pleural biopsies as well as a chemical pleurodesis in order to prevent the pleural effusion recurrence.

Furthermore, the patient reported an acute neck pain associated with tetraparesis after two weeks, during her hospital stay. A spinal MRI showed a tumoral process infiltrating the axis resulting in a spinal cord compression. There was not any sign of spinal suffering. The MRI revealed also an extended bone involvement with some nodular lesions. So, emergency decompressive radiotherapy was performed, as a salvage therapy with immobilization of the cervical spine.

**Figure 5.**
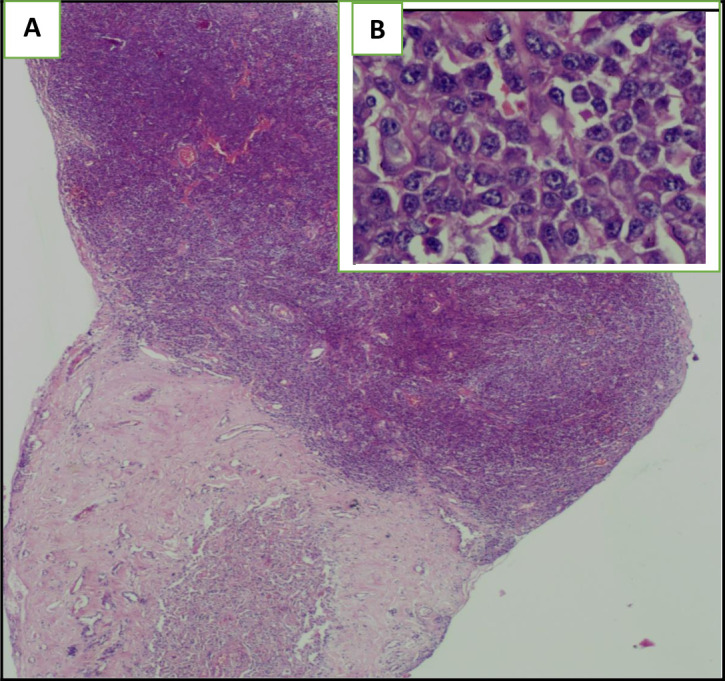
The Hematoxylin Eosin (HE) staining illustrates the presence of an undifferentiated abnormal rounded tumoral cells: (A): Envision (HE)*2, (B): Envision (HE)*40.

**Figure 6.**
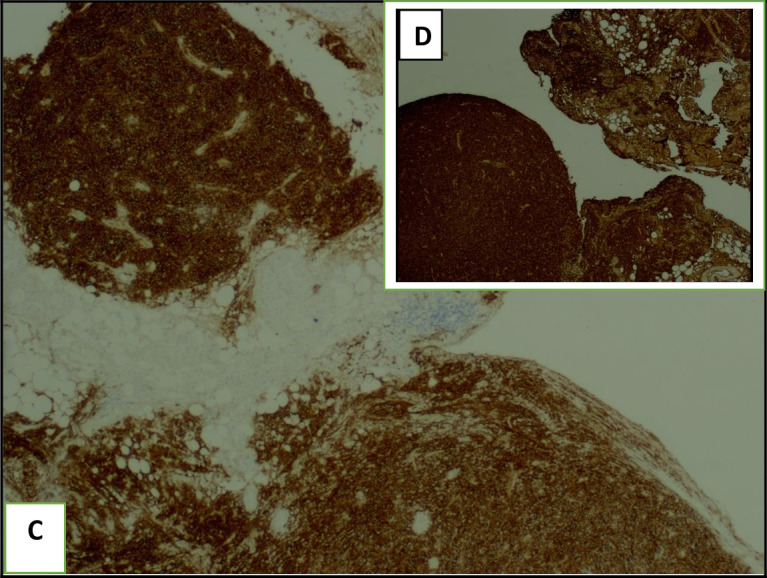
The pleural guided biopsy smear with immunofixation showed abnormal plasma cells with an intense positive CD138 staining (C) and a light kappa chain (D).

The histological examination of the pleural guided biopsy smear showed an abnormal round cell tumor ( Figure 5), with an intense and diffuse positive staining for CD138 and MUM1 ( Figure 6), associated with a kappa light chain in immunofixation suggesting a myelomatous pleural effusion (MPE). We actually noticed an increased monoclonal gamma peak (48.8 g/l) in the serum protein electrophoresis. Indeed, the immunoelectrophoresis revealed a peak in the kappa light chain region. A 24-hour proteinuria was negative. The electrophoresis and the immunofixation of urinary proteins were normal.

The diagnosis of myelomatous pleural effusion was assessed given: the presence of abnormal plasma cells with a positive staining CD 138 and MUM1 in the pleural biopsy, the presence of a kappa-light chain in the protein electrophoresis and a multiple myeloma confirmed histologically with the presence of the CRAB criteria (hypercalcemia, anemia, presence of several osteolytic lesions on imaging). Thus, the bone marrow biopsy was not necessary. Unfortunately, our patient died one month after the initial diagnosis because of a fatal progression of her disease.

## Discussion

Multiple myeloma is a bone-based disease. It was first described in Egyptian mummies. The median age of occurrence is 69 years. It occurs mainly in men. ^2^ This neoplasia can invade many organs such as the chest, the bones and the skin. However, the pleural effusions reported in these patients remain rare (6%). ^3^ Moreover, the true myelomatous pleural effusion (MPE) is extremely rare. It occurs in less than (1%) of cases. It is more common in IgG Myeloma types (40%). ^4^

The diagnosis may be challenging because the symptoms are not specific. In fact, the patients can present with a wide spectrum of clinical features. They report typically hematologic symptoms due to the bone marrow invasion (anemia, bleeding, infections) and bone lesions (hypercalcemia). They may also report symptoms due to the infiltration of the surrounding organs. ^5^^,^^6^ Our patient complained of dyspnea on exertion associated with an important deterioration of her general status.

The pleural effusion associated with multiple myeloma may be due to a congestive heart failure, mainly in secondary amyloidosis. It may result either from hyperviscosity or myocarditis, commonly reported in these patients especially after the treatment onset. ^1^^,^^7^ Pulmonary embolism should also be considered because of the advanced age of the patients, the comorbidities and the current neoplasia. ^8^ Third, the nephrotic syndrome and the chronic renal failure may be responsible of a concomitant pleural effusion. ^9^ Pulmonary embolism was ruled out in our case by a chest CT scan. The kidney function and the 24-hour proteinuria were normal. The pleural effusion may result also from a second concomitant neoplasia especially an advanced stage carcinoma (breast cancer in women, lung cancer in men) or a mesothelioma. ^1^^,^^10^ However, our patient was not a smoker and the breast exam was normal. It is worth mentioning that multiple myeloma should be considered in the differential diagnosis of a malignant pleural effusion.

The diagnosis was very challenging in our patient. In fact, we initially suspected an advanced stage lung carcinoma giving the chest CT scan’s findings, or a mesothelioma because of the multinodular aspect of the pleura in the chest ultrasound and “the grape-cluster” appearance as seen in thoracoscopy. The histological exam of the pleural guided biopsy helped us to assess the right diagnosis.

It is worth mentioning that true myelomatous pleural effusions (MPE) are extremely rare. It may result either from: a direct tumoral invasion of the pleura as seen in our patient, a direct extension from an adjacent skeletal or lung lesions, a lymphatic obstruction by compressive mediastinal lymph nodes or it may be due to the presence of an extramedullar plasmocytoma in the chest wall. ^11^^,^^12^

The diagnosis requires the presence of a monoclonal or a light chain protein in the pleural fluid, abnormal plasma cells with an intense staining CD138. ^13^^–^^16^ Medical thoracoscopy is actually recommended in case of a such malignant pleural effusion. In fact, it allows pleural guided-biopsy with a good-quality of sampling, pleural fluid drainage as well as a pleurodesis in order to avoid further pleurisy recurrence. ^8^^,^^17^^,^^18^ In our case, medical thoracoscopy showed a multinodular pleura with “a bunch of grapes” aspect. We decided to perform chemical pleurodesis using « slurry talc ». Flow cytometry may be a useful tool in the diagnosis assessement especially in these challenging cases. ^19^

Another interesting aspect of this case is that the pleural effusion revealed the disease. It is well known that the pleural involvement is a late manifestation during the natural history of the multiple myeloma often associated with a poor prognosis. ^20^ The median survival does not exceed four months according to literature, despite an aggressive therapeutic strategy. ^21^

The treatment is often based on a combination of chemotherapy drugs or a palliative radiotherapy as a salvage therapy. As regards the chemotherpay induction regimen, the VRD protocole using (Bortezomib, Lenalidomide and Dexamethasone) (VRD) is likely to offer the best risk-benefit profile. Besides, the recent introduction of target therapy using monocolonal antibodies has been a revolution in the management of these challenging cases. In fact, the 4 drug combination therapy using Daratumumab ( **DARA**)-VRD has been more efficient than chemotherapy alone with a good profile of safety. It is actually considered as the new standard treatment of an advanced stage multiple myeloma. Moreover, current clinical trials using new monoclonal antibododies such as Isatuximab (ISA) or Carfilzomib seems to be promising. In fact, the recent substitution of (Bortezomib) by a second generation proteasome inhibitor (Carfilzomib) resulted in a higher rate of sustained MRD (minimally residual disease) negativity. ^22^^,^^23^ However, these new monoclonal antibodies are not actually available in our country. Stem cell transplantation can be also considered. ^24^ Unfortunately, our patient died before the chemotherapy courses onset. Besides, the patients response are often transient despite an intensive treatment, with a high rate of death and tumoral recurrence. ^11^^,^^25^ The recent use of target therapy would improve the prognosis of these frail patients.

## Conclusion

To conclude, we reported a very rare case of a massive pleural effusion revealing multiple myeloma. It should be considered in the differential diagnosis of a malignant pleurisy. Medical thoracoscopy is a mainstream exam in the diagnosis assessement as well as in the therapeutic strategy allowing a pleurodesis in order to avoid further recurrences. New studies using flow cytometry and cytogenetic analysis are required especially in these challenging cases. The recent use of immunotherapy and target therapy seem to be very interesting pathways in the future.

## Consent

Written informed consent for publication of their clinical details and images was obtained from the family of the patient.

## Data Availability

All data underlying the results are available as part of the article and no additional source data are required.
