# Supplementary material for: Case Report: A very rare case of a Pleural Effusion revealing Multiple Myeloma
Source: F1000Res. 2023 Nov 3;12:476. Originally published 2023 May 9. [Version 3] doi: 10.12688/f1000research.133007.3 (PMC11106595; doi:10.12688/f1000research.133007.3)
Supplement: Peer review history

# Reviewer report: Reviewer response for version 3

**DOI:** 10.5256/f1000research.158236.r268152

**Published:** 2024-05-20

**Authors:** Wen Zhou

**Affiliations:** 1 Central South University, Changsha, Hunan, China

The authors presented a rare case report of multiple myeloma manifesting as massive pleural effusion. The diagnosis was confirmed by definitive pathological findings of pleural nodules, along with the presence of monoclonal protein, anaemia, and bone disease. Nonetheless, bone marrow examinations, including cell morphology, immunotyping, and Fluorescence in situ Hybridization (FISH) analysis of the multiple myeloma panel, are highly recommended. Furthermore, performing FISH analysis on tissues from pleural nodules is suggested to improve our understanding of the patient's prognosis. Given that malignant pleural effusion serves as a poor prognostic indicator, the patient died before chemotherapy. Consequently, the available information regarding the efficacy of therapeutic interventions in this condition is limited. In the discussion section, the efficacy of novel immunotherapy approaches, such as CAR-T and bispecific antibodies, are recommended to include.

Are enough details provided of any physical examination and diagnostic tests, treatment given and outcomes?

Partly

Is the case presented with sufficient detail to be useful for other practitioners?

Partly

Is sufficient discussion included of the importance of the findings and their relevance to future understanding of disease processes, diagnosis or treatment?

Yes

Is the background of the case’s history and progression described in sufficient detail?

Yes

Reviewer Expertise:

Pathogenesis and drug resistance mechanism of multiple myeloma. Malignant transformation of B cells.

I confirm that I have read this submission and believe that I have an appropriate level of expertise to confirm that it is of an acceptable scientific standard, however I have significant reservations, as outlined above.

# Reviewer report: Reviewer response for version 3

**DOI:** 10.5256/f1000research.158236.r220657

**Published:** 2023-12-13

**Authors:** Junn-Liang Chang

**Affiliations:** 1 National Defense Medical Center, Taipei City, Taiwan; 2 Pathology & Lab medicine, Taoyuan Armed Forces General Hospital, Taoyuan, Taoyuan, Taiwan

I have no comments.

Are enough details provided of any physical examination and diagnostic tests, treatment given and outcomes?

Partly

Is the case presented with sufficient detail to be useful for other practitioners?

Partly

Is sufficient discussion included of the importance of the findings and their relevance to future understanding of disease processes, diagnosis or treatment?

Yes

Is the background of the case’s history and progression described in sufficient detail?

Partly

Reviewer Expertise:

Human cancers and pathology

I confirm that I have read this submission and believe that I have an appropriate level of expertise to confirm that it is of an acceptable scientific standard.

# Reviewer report: Reviewer response for version 2

**DOI:** 10.5256/f1000research.155904.r210641

**Published:** 2023-10-25

**Authors:** Keechilat Pavithran

**Affiliations:** 1 Amrita Institute of Medical Sciences and Research Centre, Kochi, India

1. There is no mention of the M protein levels and no mention of the drugs used for the treatment. How many doses were given?
2. The regimen mentioned, VAD, is seldom used nowadays. Discussing the currently used regimens, like VRD or VCD, is better.
3. A review of the literature on all the reported cases so far will give more clarity regarding the clinical presentation and management.
4. English language editing is needed.

Are enough details provided of any physical examination and diagnostic tests, treatment given and outcomes?

Partly

Is the case presented with sufficient detail to be useful for other practitioners?

Yes

Is sufficient discussion included of the importance of the findings and their relevance to future understanding of disease processes, diagnosis or treatment?

Partly

Is the background of the case’s history and progression described in sufficient detail?

Yes

Reviewer Expertise:

Medical oncology

I confirm that I have read this submission and believe that I have an appropriate level of expertise to confirm that it is of an acceptable scientific standard, however I have significant reservations, as outlined above.

# Reviewer report: Reviewer response for version 2

**DOI:** 10.5256/f1000research.155904.r206551

**Published:** 2023-09-20

**Authors:** Junn-Liang Chang, Kuang-Ting Liu

**Affiliations:** 1 Pathology & Lab medicine, Taoyuan Armed Forces General Hospital, Taoyuan, Taoyuan, Taiwan; 2 National Defense Medical Center, Taipei City, Taiwan

The author has provided reasonable, scientific and logical explanations for the reviewer's suggestions.

Are enough details provided of any physical examination and diagnostic tests, treatment given and outcomes?

Partly

Is the case presented with sufficient detail to be useful for other practitioners?

Partly

Is sufficient discussion included of the importance of the findings and their relevance to future understanding of disease processes, diagnosis or treatment?

Yes

Is the background of the case’s history and progression described in sufficient detail?

Partly

Reviewer Expertise:

Human cancers and pathology

We confirm that we have read this submission and believe that we have an appropriate level of expertise to confirm that it is of an acceptable scientific standard.

# Reviewer report: Reviewer response for version 1

**DOI:** 10.5256/f1000research.145970.r202487

**Published:** 2023-09-07

**Authors:** Junn-Liang Chang, Kuang-Ting Liu

**Affiliations:** 1 National Defense Medical Center, Taipei City, Taiwan; 2 Pathology & Lab medicine, Taoyuan Armed Forces General Hospital, Taoyuan, Taoyuan, Taiwan

Reviewer’s comments to the authors:

1. The authors should present laboratory assays including cell blood count, PB smear analysis, and blood chemistry values, serum electrophoresis, and serum immune electrophoresis.
2. Does pleural effusion cytology show abnormal proliferation of plasma cells?
3. Has the patient had bone marrow aspiration analysis results?
4. Does the imaging test include skull, pelvis, and skeletal bones, and is there any abnormality?

Are enough details provided of any physical examination and diagnostic tests, treatment given and outcomes?

Partly

Is the case presented with sufficient detail to be useful for other practitioners?

Partly

Is sufficient discussion included of the importance of the findings and their relevance to future understanding of disease processes, diagnosis or treatment?

Yes

Is the background of the case’s history and progression described in sufficient detail?

Partly

Reviewer Expertise:

Human cancers and pathology

We confirm that we have read this submission and believe that we have an appropriate level of expertise to confirm that it is of an acceptable scientific standard, however we have significant reservations, as outlined above.
